# Case Report: Short-Term Spinal Cord Stimulation and Peripheral Nerve Stimulation for the Treatment of Trigeminal Postherpetic Neuralgia in Elderly Patients

**DOI:** 10.3389/fneur.2021.713366

**Published:** 2021-08-03

**Authors:** Lin Zhao, Tao Song

**Affiliations:** Department of Pain, The First Affiliated Hospital of China Medical University, Shenyang, China

**Keywords:** trigeminal postherpetic neuralgia, short-term stimulation, high cervical spinal cord stimulation, peripheral nerve stimulation, maxillary nerve

## Abstract

**Objective:** We aimed to report on the use of short-term high cervical spinal cord stimulation (SCS) combined with peripheral nerve stimulation (PNS) to successfully treat trigeminal postherpetic neuralgia (TPHN) affecting the V2 and V3 divisions. We also sought to use a novel PNS approach to the maxillary nerve next to the external opening of the foramen rotundum (FR) to treat TPHN at the V2 division.

**Method:** Two elderly patients successfully treated with different neuromodulation methods for TPHN are presented in this case series.

**Results:** The first case referred to an 83-year-old Chinese female patient with V2 and V3 TPHN who experienced a significant pain relief using a combination of short-term high cervical SCS at the C1–C2 level and PNS on the infraorbital nerve (ION). Case 2 was a 68-year-old Chinese male patient with V1 and V2 TPHN that obtained an excellent pain relief after having received short-term PNS on the supraorbital nerve (SON), the supratrochlear nerve (STN), and the maxillary nerve. Both reported improvements in their quality of life and ability to perform daily tasks during a 3-month follow-up period.

**Conclusions:** Short-term high cervical SCS at the C1–C2 spinal segments may be a feasible method to treat recent-onset V3 TPHN in elderly patients. Additionally, by placing the stimulation lead next to the external FR opening, we demonstrated a novel PNS approach to the maxillary nerve not previously reported for TPHN therapy.

## Introduction

Herpes zoster (HZ, or commonly known as shingles) refers to a typically vesicular rash caused by reactivation of the latent varicella zoster virus (VZV) from the sensory ganglia neurons (1). It is a viral disease characterized by skin eruptions and neuropathic pain in a specifically affected dermatome. The lifetime risk of developing HZ among the general population is approximately 10–30%, and the risk of incidence increases with older age (>50 years old) and immunosuppression (2, 3). Postherpetic neuralgia (PHN) is a refractory, long-lasting neuropathic pain complication of HZ that persists for at least 3 months after the rash onset (4). Trigeminal postherpetic neuralgia (TPHN) is a chronic severe facial pain caused by HZ and distributed in the trigeminal Gasserian ganglion or one or more branches (V1, V2, or V3) of the trigeminal nerve. TPHN pain is often described as a spontaneous, burning, or stabbing sensation and is often associated with allodynia or hyperalgesia in the damaged area, which can severely affect a patient's quality of life, physical functioning, and the ability to perform daily tasks (5). Older age is the most important factor associated with higher risk to develop to TPHN (6). Routine medical therapy for TPHN is composed of antidepressants (e.g., amitriptyline), anticonvulsants (e.g., gabapentin, pregabalin), and opioids (e.g., oxycodone, morphine) (7). However, the medication doses required to treat TPHN are frequently limited by adverse effects, such as vomiting, constipation, sedation, and drug addiction, especially in senior populations (8). Hence, finding an effective treatment to relieve TPHN in elderly patients is imperative.

Recently, some studies reported that pulsed radiofrequency (PRF) on the Gasserian ganglion might effectively treat HZ-related trigeminal neuralgia affecting any of the three branches (9, 10). However, more studies are needed to confirm its efficacy, with larger samples and a multicenter design. Peripheral nerve stimulation (PNS) is a minimally invasive treatment method that places a lead near the damaged nerve to provide direct stimulation. It has been adopted in the treatment of TPHN, primarily at the V1 or V2 areas, and seldomly at the V3 area due to the inherently high risk of lead displacement (11). Long-term high cervical spinal cord stimulation (SCS) has been successfully used to manage intractable head and facial pain, such as occipital neuralgia, V3 trigeminal neuropathic pain, and post stroke facial pain (12). This technique refers to epidural stimulation at the C1–C2 level to generate head and facial paresthesia associated with permanent implantation. However, this technique has some limitations. First, placing the percutaneous lead at the C1 or even C2 level is challenging in some individuals who underwent traumatic surgeries or had other neck lesions. Second, covering all areas of facial pain with epidural stimulation has been difficult to perform on every patient, even though the lead was adequately placed at the C1–C2 level (13). Also, some postoperative complications can severely affect a patient's quality of life and may be intolerable in elderly TPHN patients.

Here, we present two TPHN cases with uncontrolled pain following conservative measures and PRF therapy. In case 1, we attempted short-term high cervical SCS at the C1–C2 level to induce paresthesia in the lower face and jaw, combined with PNS on the infraorbital nerve (ION) to treat V2 and V3 TPHN. In case 2, we attempted short-term PNS on the supraorbital nerve (SON), the supratrochlear nerve (STN), and the maxillary nerve to treat V1 and V2 TPHN. To our knowledge, PNS on the maxillary nerve next to the external opening of foramen rotundum (FR) to treat V2 TPHN has not yet been reported.

## Case Description

### Case Report 1

**Figure 1:**
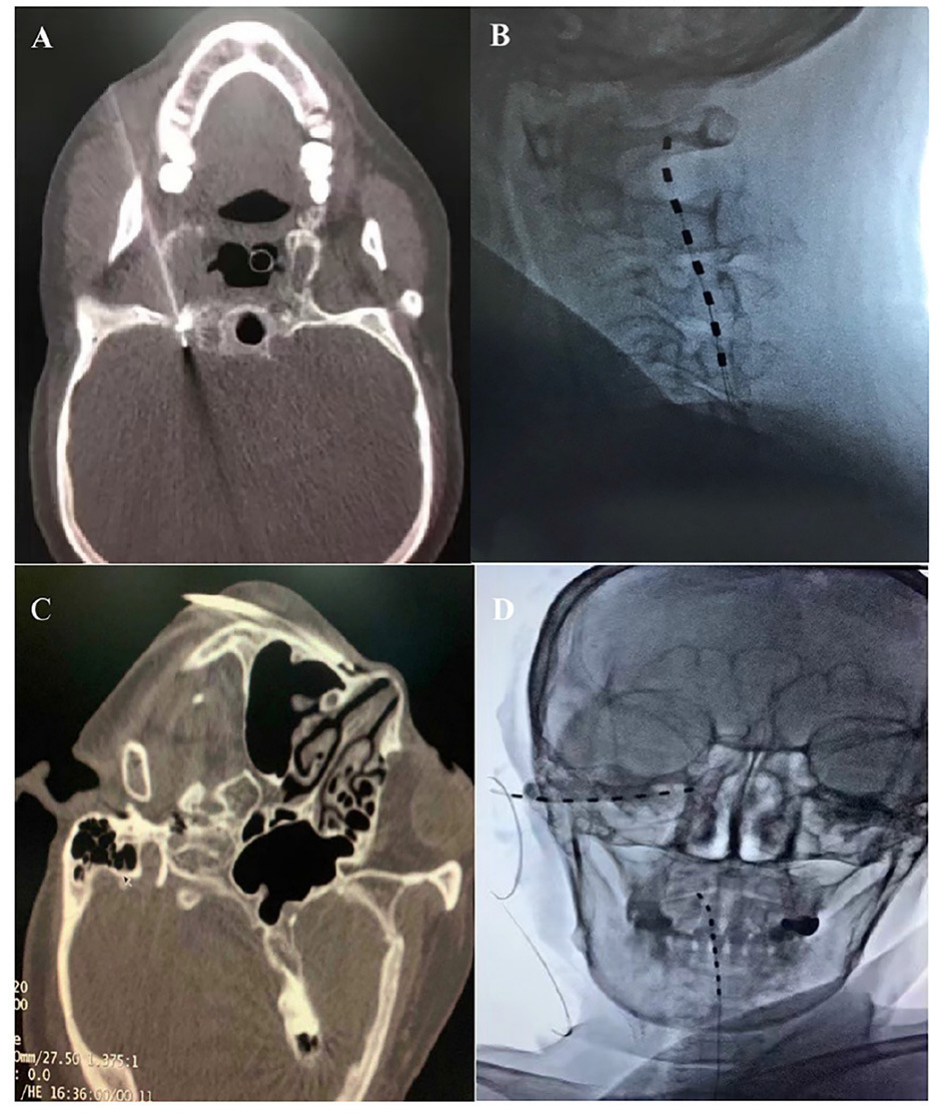
**(A)** CT-guided PRF on the Gasserian ganglion. **(B)** High cervical SCS at the C1-C2 level. **(C)** A 14-G needle inserted at the level of the right zygomatic bone. **(D)** High cervical SCS combined with infraorbital nerve (ION) PNS.

An 83-year-old Chinese female with a 4-month history of HZ-related trigeminal neuralgia since the rash onset in the V2 and V3 trigeminal divisions on the right side of the face was referred to our pain clinic. The patient denied any personal or family past medical history and displayed a severe case of spontaneous and intermittent facial pain, including the lower orbit, nose, lower jaw, lower lip, and lower teeth. A numeric rating scale (NRS) score of ≥7 on a scale between 0 and 10 was given. The patient suffered from severe paroxysmal paresthesia, including burning, shooting, and needles sensation, which increased with exposure to cold. Allodynia within the V2 and V3 territories could be triggered by a slight facial touch or brush, resulting in suboptimal sleep quality. On physical examination, the sensation to light touch and pinprick on the right V2 and V3 nerve distributions increased. The patient was medicated with gabapentin 3,600 mg and oxycodone 100 mg (equivalent to 200 mg of oral morphine) per day to manage the facial pain for 3 months without any other regular medication before coming to our pain clinic. She obtained 50% pain relief but presented with the drug-related side effects, including vomiting, constipation, and drug dependence. Upon consultation with our clinic, the patient was treated using a computed tomography (CT)-guided high-voltage, long-duration PRF on the Gasserian ganglion with a 20-G, insulated needle (14 cm,10-mm active tip, Baylis Medical Company, Montreal, Canada) (Figure 1A). The PRF treatment was performed using a Pain Management Generator (PM-230, Baylis Medical Company, Montreal, Canada), and the PRF mode was set to 42°C, a pulse width of 20 ms, and a frequency of 2 Hz for 900 s with a voltage ramp from 40 to 80 V as the procedure progressed. On the 6-day follow-up period, the patient reported definitive pain relief for the first 3 days, with the pain relief dropped to 10% on the sixth day. The reinstated pain brought back sleeplessness and a readministration of gabapentin and oxycodone. Then, the decision was made to attempt a short-term high cervical SCS. For this procedure, the patient was placed in a prone position, and T12-L1 intervertebral space was located. A 14-G needle was advanced under X-ray guidance until the epidural space was pierced. The percutaneous 1 × 8 electrode stimulation lead (Model:3189, 90 cm length, St. Jude Medical, St. Paul, MN, USA) was positioned at the high cervical region (C1–C2) (Figure 1B) immediately to the right of midline. Electrical stimulation was performed using the following settings: tonic mode (only tonic mode could be used in Chinese mainland), pulse width 450 μs, frequency 40 Hz, a constant current amplitude of 3 mA, and a contact polarity of 1– and 2+. The stimulation elicited paresthesia in the V3 division, including the lower jaw, lower lip, and lower teeth, while the pain relief of V2 division was inadequate. Thus, we attempted an additional PNS on the infraorbital nerve. The patient was then placed in a supine position with the head turned to the left. The same type of lead was positioned percutaneously through a 14-G needle from lateral to medial at the level of the right zygomatic bone (Figures 1C,D). The electrical stimulation (tonic mode, pulse width 400 μs, frequency 60 Hz, a constant current amplitude of 5 mA, and a contact polarity of 2+ and 6–) successfully covered the affected V2 facial area, including the nose and lower orbit. The patient received short-term SCS combined with PNS for 14 days with no complications. Thereafter, the leads were removed, and the patient described that individual symptoms, including spontaneous pain, needles sensation, or resting abnormal sensations were significantly improved after treatment. The NRS score dropped from 7–9 to 1–2. She was able to brush her right-sided face with cold water and had sufficient sleep quality. At her 3-month follow-up, long-lasting pain relief of 90% was sustained. The patient tapered off oxycodone entirely and maintained gabapentin on a 900-mg daily dose to relieve the remaining facial pain.

### Case Report 2

**Figure 2:**
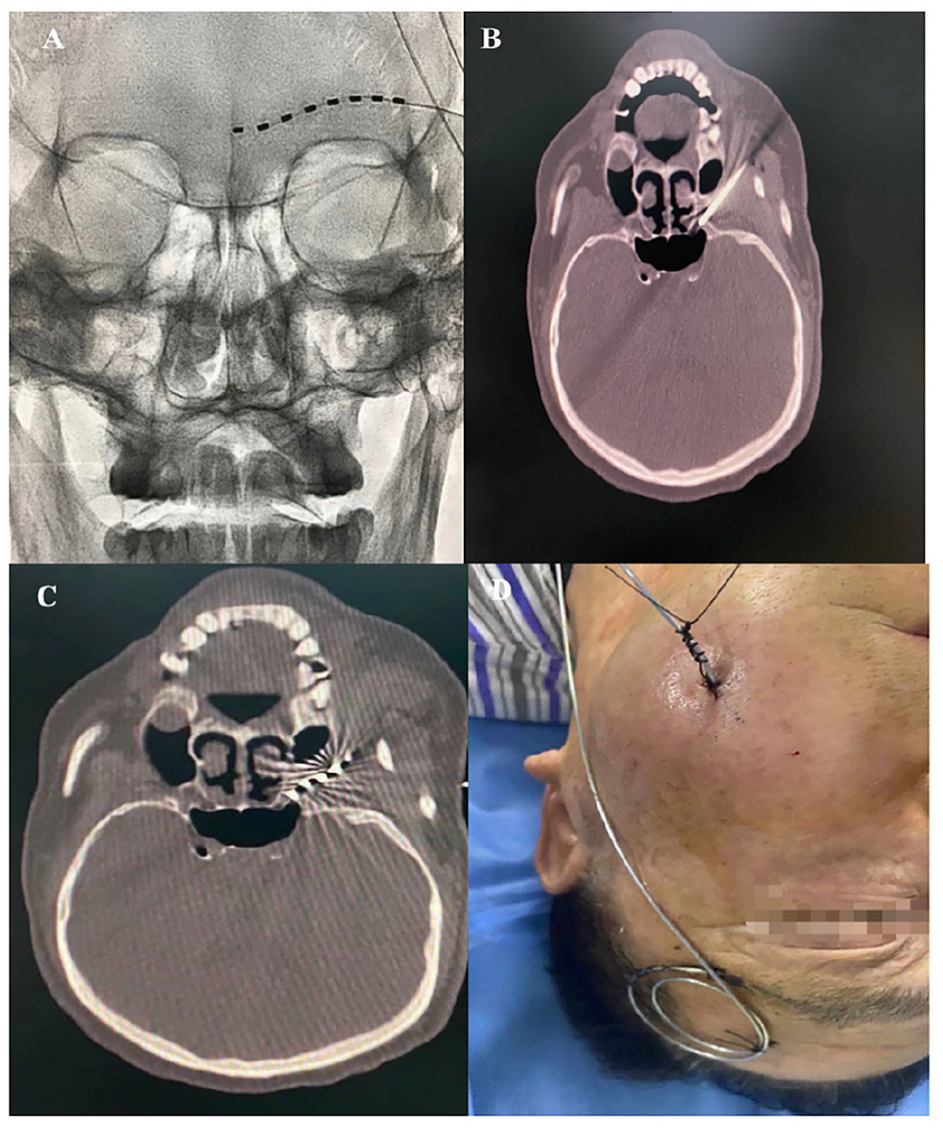
**(A)** PNS on the left SON and STN. **(B)** A 14-G needle was placed next to the left external FR opening. **(C)** The stimulation lead was placed next to the left external opening of the FR. **(D)** Both stimulation leads were anchored on the surface of the skin.

A 68-year-old Chinese male with a 5-month history of HZ-related trigeminal neuralgia since the rash onset in the V1 and V2 divisions of the left side of the face, including the forehead, upper orbit, lower orbit, nose, upper lip, and upper teeth was referred to our pain clinic. The patient had a history of ankylosing spondylitis since 2012 and was not receiving regular medication for this disease, but was instead using indomethacin (mean daily dose 100 mg) to alleviate the low back pain for 8 years. He suffered from severe spontaneous burning facial pain and numbness, especially in the areas of upper lip and upper teeth. The patient could not touch or brush his left forehead, cheek skin, and upper teeth without pain and had a NRS score between 5 and 7. On physical examination, the sensation to light touch and pinprick on the left V1 and V2 nerve distributions increased. The patient was evaluated in a chronic pain clinic and had tried medications and PRF treatments without success before coming to our pain clinic. Pregabalin 300 mg and duloxetine 120 mg/day were prescribed for 3 months with no significant pain relief. The patient was also subsequently treated with PRF on the Gasserian ganglion twice in over a 3-month period; however, he reported definitive pain relief for 14 days, then the pain relief dropped to 30%. Therefore, we attempted PNS. The patient was placed in a supine position with the head turned to the right side, and the supraorbital ridge was located. A 14-G needle was advanced under X-ray guidance from lateral to medial at the level of the left eyebrow after local anesthesia infiltration with 2 ml of 0.5% lidocaine. For the supraorbital nerve (SON) and the supratrochlear nerve (STN) stimulation, the 1 × 8 electrodes stimulation lead (Model: 3189, 90 cm length, St. Jude Medical, St. Paul, MN, United States) was positioned above the supraorbital ridge using a percutaneous needle (Figure 2A). Electrical stimulation was performed using the following settings: tonic mode, pulse width 300 μs, frequency 50 Hz, a constant current amplitude of 2 mA, and a contact polarity of 2+ and 7–. The patient obtained immediate and satisfactory pain relief in the V1 division including the forehead and upper orbit. In the hopes of providing more complete paresthesia coverage of the upper teeth painful areas, we attempted a novel PNS approach to the maxillary nerve. Then, the patient was placed in a supine position on the CT bed, and a positioning grid was placed on his left cheek. A semicoronal CT scan with a 1-mm slice thickness was conducted to locate the external foramen rotundum (FR) opening. The entry point and percutaneous puncture path were then planned. After sterilization and local anesthesia infiltration with 2 ml of 0.5% lidocaine, a 14-G needle was inserted from the inferior side of the zygomatic bone to the FR (Figure 2B). When the needle tip was confirmed to be next to the external opening of the FR on a CT scan, a second, identical stimulation lead was placed next to the external FR opening (Figure 2C). The electrical stimulation (tonic mode, pulse width 250 μs, frequency 40 Hz, a constant current amplitude of 3 mA, and a contact polarity of 1– and 2+) was performed and fully covered the affected 2nd trigeminal facial area, including the nose, lower orbit, upper lip, and upper teeth. Finally, we anchored both stimulation leads on the surface of the skin (Figure 2D). After 14 days of short-term PNS treatments, the leads were removed. No complications associated with the treatment occurred. The patient described that severe spontaneous burning pain in the previously painful areas had disappeared and numbness had been reduced by half after treatment. The NRS score dropped from 5–7 to 0–1 without any medication. He was able to brush his left face and upper teeth without any incident. During a 3-month follow-up period, long-lasting pain relief of 95–100% was sustained. The patient tapered off pregabalin and duloxetine entirely.

## Discussion

PHN is a condition that causes chronic neuropathic pain and is often associated with allodynia and/or hyperalgesia, a common complication of acute HZ infection (4). TPHN is a regional manifestation of PHN in the trigeminal ganglion. The pain can be distributed through the ophthalmic nerve (V1), maxillary nerve (V2), and mandibular nerve (V3) (14). Medical therapy is the first-line treatment for PHN. However, the adverse effects may be intolerable, especially in elderly patients. In case 1, for example, although routine 1,200 mg dose of gabapentin every 8 h and 50 mg dose of oxycodone every 12 h per os resulted in 50% pain relief, the side effects were evident and unbearable.

The Gasserian ganglion, which contains the primary sensory neuron bodies of the head and face, is chosen as a PRF target site to treat Hz-related trigeminal neuralgia (15). Wan et al. demonstrated high-voltage, long-duration PRF on the Gasserian ganglion could provide noticeable pain relief in patients with acute/subacute zoster-related trigeminal neuralgia (9). Ding et al. showed 86.7% pain relief using PRF on a Gasserian ganglion group with TPHN in a retrospective study with 1-year follow-up time (10). However, the two cases we reported underwent an identical method only to have temporary days-long pain relief before relapse. Recently, electrical stimulation of the Gasserian ganglion has been suggested to be a promising treatment modality for elderly patients with chronic intractable trigeminal neuralgia. However, Holsheimer observed a success rate of 50% in only 9% (3/34 cases) of the TPHN patients treated with the same method, and this lack of an effect was likely because pain relief by electrical stimulation is inversely related to the degree of sensory loss (16). This is also supported by the observation of Young for which the patients with TPHN had a success rate of <10% (17). Therefore, alternative therapeutic strategies were considered.

**Figure 3:**
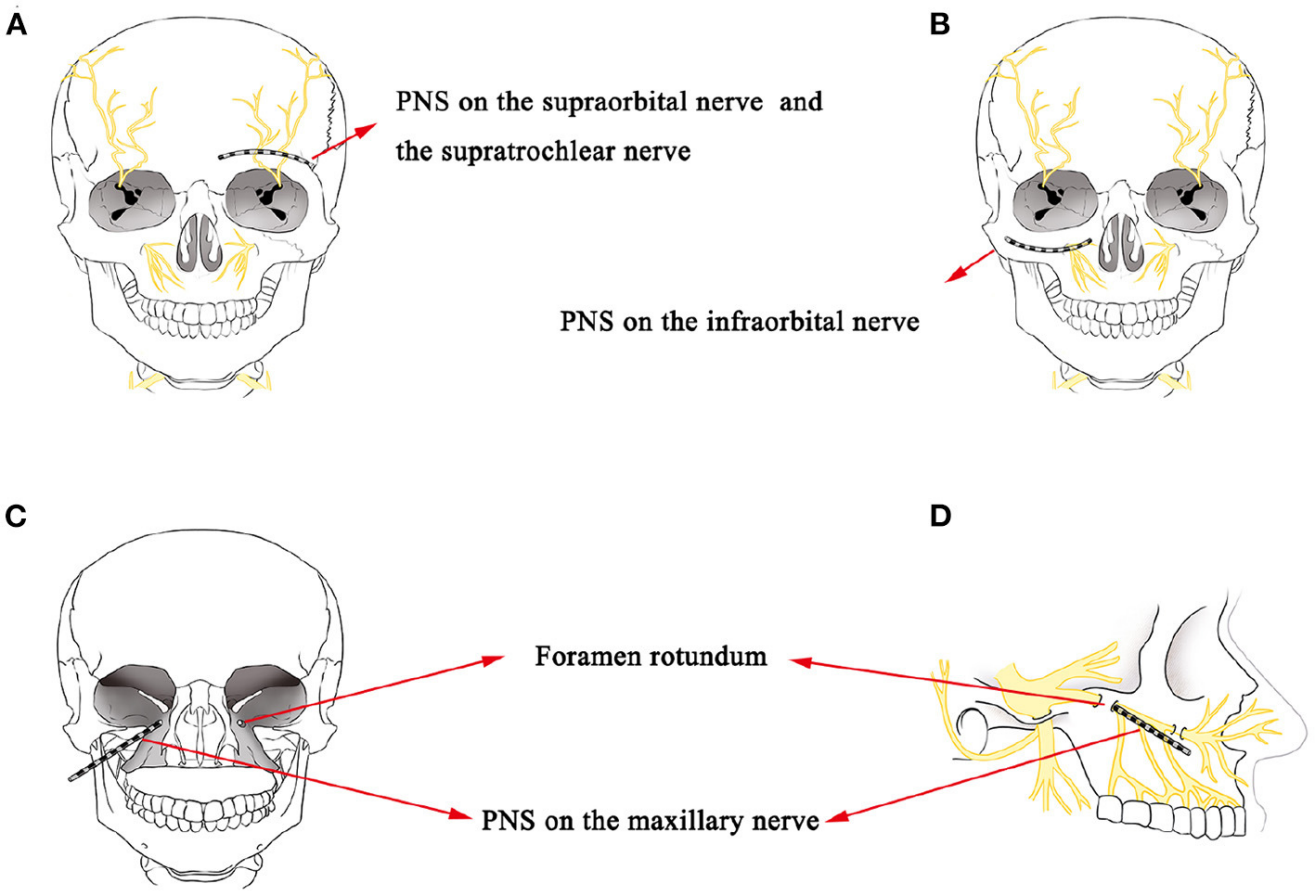
**(A)** A schematic drawing showing the stimulation lead placement for SON and STN PNS. **(B)** A schematic drawing showing the stimulation lead placement for ION PNS. **(C,D)** A schematic drawing of stimulation lead placement for maxillary nerve PNS next to the external opening of the FR.

PNS refers to the placement of a lead near a nerve to cause direct stimulation. This electrical stimulation technique has been successfully used in the treatment of HZ-related trigeminal neuralgia (18, 19). Its effect is attributed to the gate-control theory, which postulates that impulse transmissions in the nociceptive afferent pathway are modulated by the activity of large-caliber myelinated non-nociceptive A-fiber afferent nerves (11, 20). Han et al. showed that short-term PNS on the SON to treat herpes zoster ophthalmicus achieved 50% pain reduction in a 12-month follow-up without complications (18). Wan and Song also showed that 94.2% (64/68) of patients obtained excellent pain reduction during a 6-month follow-up after short-term SON PNS treatments in a retrospective study composed of 68 patients with herpes zoster ophthalmicus (19). In this case report, we treated two patients with short-term PNS on the ION in case 1 and SON and STN in case 2, respectively (Figures 3A,B), and both obtained excellent pain relief during a 3-month follow-up, consistent with the results of Han et al. and Wan and Song. Numerous PNS studies have described the placement of the leads on the SON, STN, or ION, without mentioning of placement on the mandibular nerve (18, 19, 21–25). Slavin and Wess suggested the lack of reports on using the PNS technique for the highly mobile mandibular area was due to the inherently high risk of stimulation lead displacement (11). Johnson and Burchiel also reported the same concerns regarding lead breakage caused by repeated mandibular movements (24). Therefore, finding a feasible technique to treat TPHN in the V3 area is challenging.

**Figure 4:**
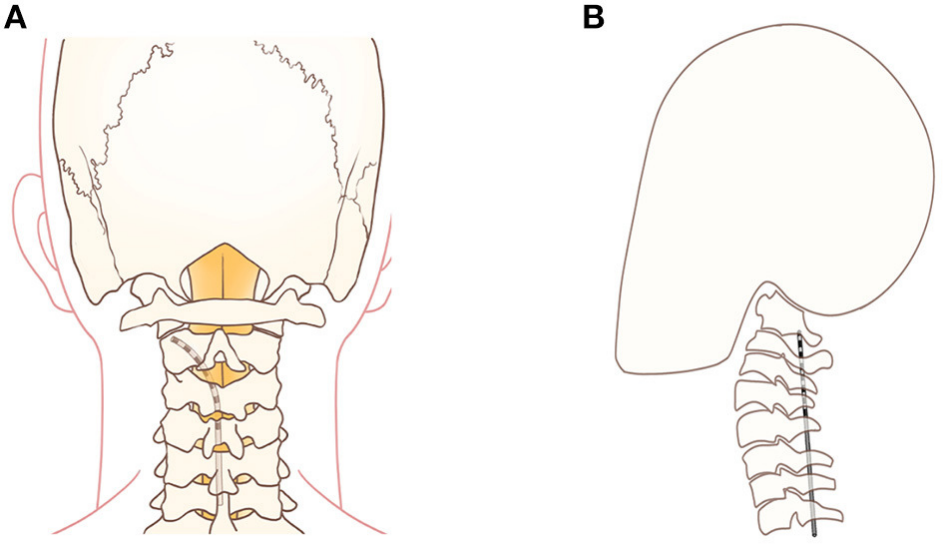
**(A)** A schematic drawing showing the stimulation lead placement for the top 2 contacts that were positioned lateral to the spinal cord midline for high cervical SCS. **(B)** A schematic drawing showing the stimulation lead placement for high cervical SCS at the level of the C1–C2 vertebrae.

SCS has been successfully used to treat HZ-related pain at the cervical, thoracic and lumbar segments (26–28). The lead is placed into the epidural space to stimulate the dorsal spinal cord columns, activate inhibitory interneurons and attenuate ascending pain transmission (29). Recently, long-term high cervical SCS has been demonstrated to be an invasive technique to treat intractable facial pain. The spinal trigeminal nucleus is the longest cranial nerve nucleus extending from the lateral medulla to the upper cervical spinal cord. The caudal sub nucleus is located laterally to the dorsal columns and is the primary trigeminal afferent transmission site for the mandibular nociceptive neurons and is the site that high cervical SCS neuromodulates (30, 31). Therefore, when the lead is placed laterally away from the medial dorsal columns, high cervical SCS could be more effective for treating facial pain (31). On the other hand, when using SCS to treat PHNs in the neck, chest, and waist positions, the leads are placed on the dorsal column of the spinal cord. When treating TPHN, the lead should be placed at the C1–C2 level and on the outside of the dorsal column of the spinal cord to induce V3 area paresthesia. In case 1, we attempted percutaneous high cervical SCS to stimulate the nucleus caudalis. The lead was placed at the C1–C2 level (Figure 4B). The top 2 lead contacts were manipulated to be in a position that was lateral to the spinal cord midline in the cervical epidural space (Figure 4A) with a contact polarity of 1– and 2+ to successfully evoke a sense of paresthesia instead of pain in the V3 division, including the lower jaw, lower lip, and lower teeth. Finally, full facial pain coverage (V2 and V3 divisions) was achieved and provided diffuse analgesic effects using the short-term ION PNS and the high cervical SCS. Long-term high cervical SCS to treat intractable head or facial pain has been proved effective in some studies. Velásquez et al. reported on 12 patients with trigeminal neuropathic pain that were treated with upper cervical SCS trials. All of them (one had TPHN) received permanent implantations by placing the leads on the craniocervical junction and obtained significant pain relief with a postoperative NRS score of 3 compared with a baseline of 7 (32). However, seven patients had a total of 19 revised procedures, and three patients were identified as therapy failures during a mean 4.4-year follow-up period. Chivukula et al. reported on 25 of 36 patients with facial pain (four had TPHN) that were treated with high cervical SCS and permanently implanted lead placements in the cervicomedullary junction. These patients averaged 50% pain reduction during a mean 3.9-year follow-up period (33). Tomycz et al. reported on 16 of 25 patients with facial pain (two had TPHN) that were treated with high cervical SCS and permanently implanted lead placements in the cervicomedullary junction, and 75% (12/16) of the patients reported significant improvements in quality of life during a mean 53.4-month follow-up period (12). All three authors surgically exposed the epidural space and implanted leads in the cervicomedullary junction. However, complications such as surgical site infections and lead migrations can occur. Conversely, in our study, we adopted a reversible, minimally invasive technique of using short-term high cervical SCS to treat V3 TPHN, and the patient obtained significant pain relief with no complications after 14 days of stimulation and pain relief persisted at least through the 3-month follow-up period. Characteristics and results of studies that have assessed the use of pulsed radiofrequency, peripheral nerve stimulation, and high cervical spinal cord stimulation to treat HZ-related and other types of facial pain have been shown in Table 1.

**Table 1:** Characteristics and results of studies that have assessed the use of pulsed radiofrequency, peripheral nerve stimulation, and high cervical spinal cord stimulation to treat HZ-related and other types of facial pain.

**Reference**	**Publish year**	**Study design**	**Diagnosis**	**Sample**	**Pain distribution**	**Location**	**Therapeutic modality**	**Characteristics of the intervention**	**Follow-up**	**Pain outcomes**	**Complications**
Wan et al. (9)	2019	RCT	Acute/Subacute zoster-related trigeminal neuralgia	*n* = 96	V1, V2, V3	Gasserian ganglion	PRF	PRF group (*n* = 48): 42°C, 2 Hz, 20 ms for 900 seconds and electric voltage (60–90 V) Sham group (*n* = 48): no radiofrequency energy	6 months	VAS scores and the average dosage of pregabalin administered in the PRF group were significantly lower than Sham	Ecchymoma (PRF/Sham:7/14)
Ding et al. (10)	2019	R	TPHN	*n* = 90	V1, V2, V3	Group A (*n* = 45): peripheral nerve Group B (*n* = 45): gasserian ganglion	PRF	42°C for 300 s	12 months	Total efficiency : group A/group B:68.8% (31/45)/86.7% (39/45)	No report
Han and Song (18)	2020	R	HZO	*n* = 18	V1	SON	PNS	Short-term (*n* = 18)	6 months (*n* = 18) 12 months (*n* = 11)	VAS: Baseline:7.3 ± 0.9 (*n* = 18) 6 months: 2.6 ± 0.9 (*n* = 18) 12 months:2.1 ± 0.9 (*n* = 11)	None
Wan et al. (19)	2020	R	HZO	*n* = 68	V1	SON	PNS	Short-term (*n* = 68)	6 months	94.2% (64/68) patients obtain excellent pain relief (NRS ≤ 2)	None
Texakalidis et al. (21)	2020	R	Facial pain	*n* = 15: post-traumatic (*n* = 5) idiopathic (*n* = 7) post-herpetic (*n* = 2) post-stroke (*n* = 1)	V1,V2	SON,ION	PNS	Permanent implantantion (*n* = 12) post-traumatic (*n* = 4) idiopathic (*n* = 5) post-herpetic (*n* = 2) post-stroke (*n* = 1)	Median: 5.8 months	Median pre/post-operative VAS: 7/1.8	Lead erosions (*n* = 1)
Lerman et al. (22)	2015	C	TPHN	*n* = 1	V1	SON, STN	PNS	Permanent implantantion (*n* = 1)	9 months	pre/last VAS: 8-10/1	None
Stidd et al. (23)	2012	C	TNP	*n* = 3: Pt 1: TNP secondary to enucleation Pt 2: TNP secondary to zygomaticomaxillary fracture Pt3: TPHN	V1, V2	SON, ION	PNS	Permanent implantantion (*n* = 3)	Pt1: 27 months Pt2: 23 months Pt3: 6 months	Pain relief: Pt 1: 100%Pt 2: 100% Pt 3:60%	Pt 3: lead migration
Johnson and Burchiel (24)	2004	R	TNP	*n* = 10: Traumatic superaorbital neuralgia (*n* = 3) Traumatic infraraorbital neuralgia (*n* = 2) Postherpetic superaorbital neuralgia (*n* = 4) Refractory trigeminal neuralgia (*n* = 1)	V1, V2	SON, ION	PNS	Permanent implantation (*n* = 10)	Mean: 26.6 ± 4.7 months	70% (7/10) patients experienced at least 50% pain relief and a decrease in medication use	Wound breakdown (*n* = 2) Short extension cable (*n* = 1)
Dunteman (25)	2002	C	TPHN	*n* = 2	V1	SON	PNS	Permanent implantation (*n* = 2)	Pt 1: 36 months Pt 2: 36 months	Pt1: complete resolution of the burning pain above the eye and in the forehead region. Mild aching remained in the scalp region at a 4/10 VAS level Pt 2: stop using daily ice packs, discontinue methadone, and diminish oxycodone use by greater than 50%	None
Velásquez et al. (32)	2018	R	TNP	*n* = 12. Post-traumatic (*n* = 3) Post-herpetic (*n* = 1) TNP (*n* = 8)	V1, V2, V3	Craniocervical junction	Upper cervical SCS	Permanent implantation (*n* = 12)	Mean:52.8 months	median baseline/trial/postoperative NRS:7/3/3 7 patients performed on 19 revision procedures 3 patients were identified as therapy failures	Postoperative infection (*n* = 1)
Chivukula et al. (33)	2014	R	Facial pain	*n* = 36: TDP (*n* = 14) TNP (*n* = 7) PHN (*n* = 4) ON (*n* = 11)	V1, V2, V3	Cervicomedullary junction	High cervical SCS	Permanent implantation (*n* = 25) TDP (*n* = 10) TNP (*n* = 4) PHN (*n* = 4) ON (*n* = 7)	Mean: 46.8 months	Mean more than 50% pain reduction	Infection (*n* = 2) Lead migration (*n* = 5)
Tomycz et al. (12)	2011	R	Facial pain	*n* = 25 TDP (*n* = 10) TPN (*n* = 5) PHN (*n* = 2) ON (*n* = 7) Post-stroke (*n* = 1)	V1, V2, V3	Cervicomedullary junction	High cervical SCS	Permanent implantation (*n* = 16) TDP (*n* = 7) TPN (*n* = 4) PHN (*n* = 2) ON (*n* = 2) Post-stroke (*n* = 1)	Mean: 53.4 months	75% (12/16) patients report significant improvement in quality of life	Infection (*n* = 1)

*RCT, Randomized Controlled Trial; R, Retrospective Study; C, Case Report; HZO, Herpes Zoster Ophthalmicus; TPHN, Trigeminal Postherpetic Neuralgia; TNP, Trigeminal Neuropathic Pain; TDP, Trigeminal Deafferentation Pain; PHN, Postherpetic Neuralgia; ON, Occipital Neuralgia; ION, Infraorbital Nerve; SON, Supraorbital Nerve; STN, Supratrochlear Nerve; PRF, Pulsed Radiofrequency; PNS, Peripheral Nerve Stimulation; SCS, Spinal Cord Stimulation; P, Patient; NRS, numeric rating scale; VAS, visual analog scale*.

In the second case, we attempted a novel PNS approach directly to the maxillary nerve next to the external opening of the FR to treat V2 TPHN in the hopes of providing more complete paresthesia coverage of the maxillary region (Figures 3C,D). According to the anatomical structure, the maxillary nerve stems from the Gasserian ganglion and passes through the FR (34). It then divides into several branches, including the ION, the superior alveolar nerve, the zygomatic nerve, and the nerves of the sphenopalatine ganglion. These branches provide sensory innervations to the lower eyelid, nose, cheek, upper lip, and upper teeth (35). The FR is regarded as a critical anatomic structure for locating the maxillary nerve. The key for a successful maxillary nerve stimulation pertains to placing the stimulation lead near the maxillary nerve next to the external opening of the FR. Traditional ION PNS is a minimally invasive treatment method that places a lead near the ION to provide direct stimulation (Figure 3B). The ION is a continuous branch of the maxillary nerve and is responsible for sensory innervation to the lower eyelid, nose, upper lip, and part of teeth, including upper incisors, canines, premolars, and the root of the first molar (36). According to our previous clinic experience, some patients who underwent ION PNS therapy had incomplete paresthesia coverage over the upper teeth, especially at the second and third molars, which are innervated by the posterior superior alveolar nerve, additional branches of the maxillary nerve (37). Therefore, compared with the traditional ION PNS, the maxillary nerve trunk was directly targeted instead of targeting the maxillary nerve branches with this novel stimulation lead placement and provided more complete paresthesia coverage of the maxillary region. The novel placement of the lead near the maxillary nerve could be one factor as to the excellent pain relief achieved in the areas of upper teeth for the second patient of this report.

Long-term SCS or PNS have been used to treat refractory PHN (38), and most authors have adopted permanent implantation methods. However, are these invasive procedures necessary to treat early PHN in elderly patients? In this report, we presented two elderly patients treated 1 and 2 months after the onset of TPHN, respectively, that were considered to be in the early stages of PHN, defined as pain persisting longer than 3 months. Both patients obtained persistent and excellent pain relief, a reduction in the need for analgesics, and improvement in quality of life after 14-day short-term SCS and PNS treatments over a 3-month follow-up period. The results indicated that short-term neuromodulation therapy may be effective in improving pain and reducing medication requirement for early TPHN patients. The extended pain relief effect could be explained by a reversal in peripheral and central sensitization during the early neuropathic pain stage. It is generally believed that the development and maintenance of neuropathic pain induced by nerve injury is associated with increased sensitivity and excitability of primary sensory neurons in the peripheral nervous system and nociceptive neurons in the spinal cord and brain in the central nervous system (39). Researchers have observed that electrical stimulation of the dorsal column did not only attenuate dorsal horn neuronal excitability but inhibited spinal wide-dynamic-range neuronal activity in neuropathic pain models (40). Our previous study showed that performing short-term SCS during the early stages of PHN could help prevent the development of pain hypersensitivity and reverse the development of peripheral and central sensitization (28). Stevanato et al. reported that PNS could affect changes in nociceptive signals that normalized peripheral and central sensitization leading to a reduction of allodynia (41). Yanamoto and Murakawa reported that 63.6% (21/33) of patients achieved a significant pain relief and showed that short-term SCS was an effective treatment for early PHN within 1–6 months of its onset (42). Together, these results showed that short-term SCS and PNS could be an effective method for elderly patients with early PHN. Although it is possible that early PHN may be relieved spontaneously without short-term SCS and PNS interventions, the two patients of this report with intractable TPHN were resistant to both drug and PRF therapies, and their NRS scores were as high as 9 and 7 after 4 and 5 months, respectively, from the onset of the disease. Also, compared with the cost of short-term SCS or PNS, which is approximately equivalent to US$2,000 in the Chinese medical system, the cost of an impulse generator is approximately equivalent to US$25,000. This amount of money would cause a massive financial burden for elderly patients. Therefore, for all of the reasons stated above, short-term neuromodulation therapy could be considered a promising therapy for elderly patients with early PHN.

## Study Limitations

There were several limitations in this case series, which should be addressed in future research. First, this was a retrospective study with small sample size and short follow-up time. Our results should be regarded as preliminary data, and prospective randomized clinical trials with large sample sizes and long-term follow-up time are needed to validate these results. Second, the majority of included studies in the discussion were retrospective studies or case reports with small sample size. High quality of evidence such as randomized controlled trials are needed to further assess the treatment effects of short-term SCS and PNS for TPHN. Third, both reported cases had relatively short-term follow-ups. Since, the proposed effect of a short-term central or peripheral stimulation would be to inhibit the development of pain sensitization, and therefore persistent pain, further studies should asses the efficacy of these interventions in the long term.

## Conclusions

Short-term high cervical SCS at the C1–C2 spinal segments may be a feasible method to treat recent-onset V3 TPHN in elderly patients, which is challenging when using conventional therapies. PNS on the maxillary nerve next to the external opening of the FR to treat V2 TPHN is a novel approach which has not been reported previously. Compared with the traditional ION PNS, we theorized that this novel approach would provide more complete paresthesia coverage of the maxillary region.

## Data Availability Statement

The raw data supporting the conclusions of this article will be made available by the authors, without undue reservation.

## Ethics Statement

The studies involving human participants were reviewed and approved by The Ethics Committee of the First Affiliated Hospital of China Medical University. The patients/participants provided their written informed consent to participate in this study. Written informed consent was obtained from the individual(s) for the publication of any potentially identifiable images or data included in this article.

## Author Contributions

All authors listed have made a substantial, direct and intellectual contribution to the work, and approved it for publication. LZ is responsible for case writing and literature indexing. TS is responsible for case summary and full text guidance.

## Conflict of Interest

The authors declare that the research was conducted in the absence of any commercial or financial relationships that could be construed as a potential conflict of interest.

## Publisher's Note

All claims expressed in this article are solely those of the authors and do not necessarily represent those of their affiliated organizations, or those of the publisher, the editors and the reviewers. Any product that may be evaluated in this article, or claim that may be made by its manufacturer, is not guaranteed or endorsed by the publisher.
